# Brains in Sync: Practical Guideline for Parent–Infant EEG During Natural Interaction

**DOI:** 10.3389/fpsyg.2022.833112

**Published:** 2022-04-27

**Authors:** Elise Turk, Yaara Endevelt-Shapira, Ruth Feldman, Marion I. van den Heuvel, Jonathan Levy

**Affiliations:** ^1^Department of Cognitive Neuropsychology, Tilburg University, Tilburg, Netherlands; ^2^Baruch Ivcher School of Psychology, Interdisciplinary Center Herzliya, Reichman University, Herzliya, Israel; ^3^Department of Neuroscience and Biomedical Engineering, Aalto University, Espoo, Finland

**Keywords:** brain-to-brain synchrony, dual-EEG, EEG, hyperscanning, parent–infant interaction, neural synchrony

## Abstract

Parent–infant EEG is a novel hyperscanning paradigm to measure social interaction simultaneously in the brains of parents and infants. The number of studies using parent–infant dual-EEG as a theoretical framework to measure brain-to-brain synchrony during interaction is rapidly growing, while the methodology for measuring synchrony is not yet uniform. While adult dual-EEG methodology is quickly improving, open databases, tutorials, and methodological validations for dual-EEG with infants are largely missing. In this practical guide, we provide a step-by-step manual on how to implement and run parent–infant EEG paradigms in a neurodevelopmental laboratory in naturalistic settings (e.g., free interactions). Next, we highlight insights on the variety of choices that can be made during (pre)processing dual-EEG data, including recommendations on interpersonal neural coupling metrics and interpretations of the results. Moreover, we provide an exemplar dataset of two mother–infant dyads during free interactions (“free play”) that may serve as practice material. Instead of providing a critical note, we would like to move the field of parent–infant EEG forward and be transparent about the challenges that come along with the exciting opportunity to study the development of our social brain within the naturalistic context of dual-EEG.

## Introduction

Starting from birth, dyadic interaction between parent and infant shapes the developing social brain (Feldman, 2012, 2021; Bell, 2020). Optimal parental interactions can be defined as a *synchronous relationship* in which the parent is attuned to the infant’s moment-by-moment state and social signals and provides stimulation accordingly (Feldman, 2007b). These interactions contain, by definition, episodes of matching alongside moments of non-matching (for instance, the infant is attentive and the mother is socially stimulation) or moments of mismatch and reparation (Tronick and Cohn, 1989); still, the entire social episode is coordinated and synchronous. The theoretical *synchrony model* indicates that optimal interactions between parents and infants serve as a regulatory function to the infant’s emerging social abilities and neurobiological states (Feldman, 2007b,^2016^; Carollo et al., 2021). Several decades of research have shown that behavioral synchronization is indeed a key feature of parent–infant interaction that has long-term effects on socialization and empathy, emotion regulation, mental health, and maturation of the social brain across childhood, adolescence, and up until adulthood (Feldman, 2007a,^2010^; Leclere et al., 2014; Levy et al., 2017, 2021). In parallel, disruptions to the synchronous relationship between parent and child, due to conditions such as maternal postpartum depression or child early life stress, carry long-term negative consequences for social development and the social brain (Pratt et al., 2017, 2019; Levy et al., 2019; Feldman, 2020). Disruptive synchronous interactions typically come in two forms; those with reduced sensitivity of the parent with no temporal contingencies between the infant’s communication and the parent’s response and those in which the parent is intrusive and overstimulating, disregarding the infant’s signal for rest and regrouping (Cohn and Tronick, 1988; Feldman et al., 2009; Granat et al., 2017). More recent studies have shown that synchrony between parent and infant can be observed across neurobiological systems, including parent–infant synchrony of heart rhythms (Feldman et al., 2011) or oxytocin response (Feldman et al., 2010a), which are accompanied by behavioral synchrony (for review see, Feldman, 2020). Only recently, empirical studies using a parent–infant hyperscanning paradigm began to show that adult’s and infant’s *neural* activity is also synchronized during social interaction (see for review: Markova et al., 2019; Wass et al., 2020; Turk et al., 2022).

In developmental research, hyperscanning comprises the simultaneous measurement of brain activity in parent/adult and child using different neuroimaging methods, including dual-EEG (Atzaba-Poria et al., 2017; Leong et al., 2017; Krzeczkowski et al., 2020; Endevelt-Shapira et al., 2021; preprint Wass et al., 2018; Leong et al., 2019; Perone et al., 2020; Santamaria et al., 2020), dual-fNIRS (Reindl et al., 2018; Azhari et al., 2019; Miller et al., 2019; Nguyen et al., 2020, a,b,c; Piazza et al., 2020; Quinones-Camacho et al., 2020; Wang et al., 2020) and dual-MEG (Hirata et al., 2014; Hasegawa et al., 2016) or sequential-MEG (Levy et al., 2017, 2021). While all these studies clearly demonstrate that social interactions are marked by the coordination of two brains, so called *brain-to-brain synchrony*, the empirical methods used in these studies show great variability (Czeszumski et al., 2020). As compared to MEG, EEG and fNIRS are the most mobile and infant-friendly systems. One of the main differences between EEG and fNIRS, is that they measure different types of neural activity. While EEG measures the electrical activity of the brain, fNIRS measures changes in BOLD signal. As a results, the two methods have different temporal and spatial properties (e.g., respectively, fast versus slow neural responses and, spatially more general versus specific to certain cortical regions), and therefore answer different aspects of neurobehavioral attunement of social partners (Hamilton, 2021). Moreover, EEG facilitates the opportunity to reduce the amount of movement-related artifacts, while fNIRS has a much better tolerance to movements due to the compatibility with motion sensors (Lloyd-Fox et al., 2010). Recently, a complete guide of parent-child (i.e., hyperscanning) fNIRS data processing and analysis is developed to accommodate the growing need for transparency and replicability in performing fNIRS studies (Hoehl et al., 2021; Nguyen et al., 2021a).

Here, we will focus on parent–infant EEG as it enables the exploration of synchrony at the level of brain rhythms and importantly because it is a well-adapted infant-friendly method to tackle brain-to-brain synchrony at the speed of fast changing social interaction in a natural setting. Parent–infant EEG studies provided accumulating evidence for the coupling of neural activity and attunement of fast-changing social signals such as attention (Wass et al., 2018), mutual gaze (Leong et al., 2017; Endevelt-Shapira et al., 2021), negative behavior (Atzaba-Poria et al., 2017), and emotions (Krzeczkowski et al., 2020; Perone et al., 2020; Santamaria et al., 2020) between parent and infant. These fascinating initial findings underscore the multitude of possibilities to investigate parent–infant synchrony on a whole new multimodal and interpersonal level (for review see, Markova et al., 2019; Wass et al., 2020; Turk et al., 2022), making it possible to explore new neurobiological aspects of natural interaction and to examine differences in clinical cohorts (Leong and Schilbach, 2019).

The number of studies that simultaneously measure EEG-based brain activity during parent–infant interaction are rapidly growing. However, common grounds on how to acquire the data and estimate brain-to-brain synchrony as well as the validity of the method are still largely lacking (Burgess, 2013; Turk et al., 2022). Consequently, instead of getting new insights in underlying interpersonal neural interactions, this variety may make it hard to compare studies and may lead to problems of reliability and validation of synchronization (Burgess, 2013; Hamilton, 2021). There are numerous methodological issues in the dual-EEG field and in parent–infant EEG in particular. Multiple efforts are taken by hyperscanning-researchers to overcome challenges by sharing their experiences and expert opinions. So far, hyperscanning-researchers already provided an overview of experimental setups of dual-EEG (Barraza et al., 2019), the HyPyP pipeline for analyzing dual-EEG data to compute (various forms of) brain-to-brain synchrony (Ayrolles et al., 2021), and, very recently, the DEEP pipeline for the (pre)processing of developmental dual-EEG (Kayhan et al., 2022). Additionally, comprehensive expert opinions on hyperscanning sensitivity analyses (Burgess, 2013), mechanistic interpretations of brain-to-brain synchrony (Hamilton, 2021), infant EEG and data loss (Stets et al., 2012; Cuevas et al., 2014; van der Velde and Junge, 2020), practical aspects of infant ERP (Hoehl and Wahl, 2012), movement artifacts in infant and adult EEG (Georgieva et al., 2020), and the challenges and solutions of EEG research with infants in social experiments (Noreika et al., 2020), tackle a lot of challenges that dual-EEG and infant EEG researchers may encounter and move the field forward. Yet, thus far, a comprehensive guide specifically for parent–infant EEG is missing from the literature.

Parent–infant EEG is an exciting novel technique, and we would like to address the growing need of researchers to have a well-documented overview of the possibilities and requirements of a parent–infant EEG experiment. The goal of this review is to assist in developing parent–infant EEG experiments by guiding researchers through *all* aspects of the research: *equipment, data collection, preprocessing*, *analyzing* parent–infant EEG, and data *interpretation*. In this guide, we provide practical tips for the experiment from our own experiences and examples from the literature. It is important to emphasize that there is no consensus on the best practices of parent–infant EEG (or dual-EEG in general) yet. Consequently, we rather provide an overview of different practical possibilities without suggesting superiority of one method over the other. Additionally, we included an open-access example of a mother–infant EEG dataset (*N* = 2) that may help to explore the various forms of brain-to-brain synchrony during natural interaction and test out preprocessing pipelines before data collection.

## Experimental Equipment and Design

### Parent–Infant EEG Equipment

**FIGURE 1:**
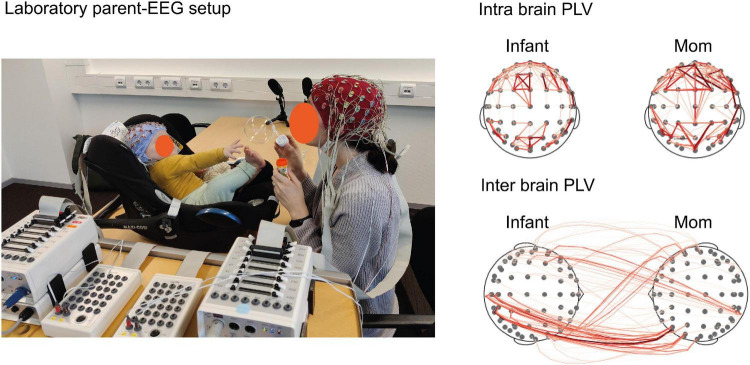
Parent–infant EEG setup and exemplary data. ‘*Lab setting’* on the **(Left)**, image is showing our lab’s parent–infant dual-EEG setup using BioSemi ActiveTwo. The infant is placed in a baby car seat on top of the table which enables the direct eye contact between parent and infant. The parent (red) and infant (light blue) caps with 64 electrodes (installed after cap placement) are each connected to a separate AD-Box that is interconnected with the other AD-Box. For technical details of the BioSemi setup, see Barraza et al. (2019). For this explanatory guideline we used the *free play* task as paradigm of 3 min, while recording dual-EEG, video and audio. EEG measures during free play represents the brain state of natural interaction between parent and infant. *‘Intra brain and inter brain visualization’* on the **(Right)**, image is showing PLV connectivity based on infant Alpha (6–9 Hz). The visualizations are based on one dyad from the (freely available) dataset for this study including the data of 2 mother–infant dyads and are analyzed in HyPyP. Infant’s age was 11 months. The connections (red lines) represent positive significant associations, the thickness of the lines represents the connectivity strength (PLV values transformed to Cohen’s *D*, threshold = 2). Dyads were included from Tilburg University’s Brains-In-Sync cohort (PI: van den Heuvel). *Privacy note*: the neural data does not correspond to the mom and infant on the image.

The laboratory setup must be compatible with two connected EEG systems to be able to acquire simultaneous measurement of brain activity in parent and infant. It is challenging to transform an EEG laboratory setup - that is usually designed for single-adult experiments – to a parent–infant EEG setup. Practical steps that can be taken to build a laboratory setup for dual-EEG experiments are extensively described by Barraza et al. (2019). In this methodological overview, the authors (including our group) provide implementations of EEG setups, allowing independent measures of each individual and measures of synchronization between the signals of two different brains, using hardware and software from different companies (including Brain Products, ANT, EGI, and BioSemi) (Barraza et al., 2019). With minor adaptations, this protocol can easily be translated to a parent–infant EEG experiment. In short, the EEG box/amplifier of the infant has to be connected to the adult box/amplifier to allow savings of both recordings into one file. The way this is accomplished varies between EEG systems used. Figure 1 displays the experimental dual-EEG setup with hardware from Biosemi (ActiveTwo AD-box with extra input socket, called a “daisy-chain,” Biosemi, Amsterdam, Netherlands) that has been used by our group. The 64-electrode caps of mom and infant are connected to separate boxes with different speed modes, to identify the different EEG datasets. The boxes are connected in series and one of the two boxes (the ‘master’ box) is connected to the computer and sends both datasets to the software (e.g., ActiView). More technical details can be found in the methods paper of Barraza et al. (2019).

Besides the stationary EEG systems, mobile (i.e., portable) EEG systems are gaining ground in outside-the-laboratory research with children (Giannadou et al., 2022; see for review, Lau-Zhu et al., 2019), infants (Troller-Renfree et al., 2021) and in EEG hyperscanning (i.e., dual-EEG or EEG with more than one social partners) studies (Dikker et al., 2017, 2021). There are - as far as we are aware – no dual-EEG studies with infants that use mobile EEG systems, but it would be a major opportunity for future purposes. Portable systems have no cables hanging around that could distract the infant, it can be used outside the laboratory, it allows more freedom of movements (Lau-Zhu et al., 2019) and it is possible to track head motion at the same time (see for example, Straetmans et al., 2022). However, mobile EEG systems have their own challenges; they often have fewer electrodes, dry-type electrodes (without gel), and can suffer from connectivity issues (not saving data).

For parent–infant EEG, you need an age-specific EEG cap for the infant with the same number of electrodes on the same topological space as the adult cap, including (for some systems) multiple infant-friendly online and offline reference electrodes that define common ground in the adult and infant. Different reference electrodes have their advantages and disadvantages and which reference electrode options you have are also dependent on the system/caps that you are using. An overview of different dual-EEG setups can be found in Barraza et al. (2019). In the ActiveTwo setup we are using a combination of CMS (Common Mode Sense) and DRL (Driven Right Leg), which are online reference electrodes or so-called ground electrodes that are built in the cap of the ActiveTwo system, and electrodes that are placed on the mastoids (M electrodes) for offline referencing. Electrodes on the mastoids seem to be picking up the most trustworthy ground signal, as they are placed on the bones next to the ears. However, placement on a moving infant can be difficult and removing the adhesive tape can be painful (due to hair) if not executed well (Noreika et al., 2020). In our experience, well trained experimenters can place and remove the mastoid electrodes without causing distress in the infant. Cz (or FCz-electrode on top of the head) is the most commonly used child-friendly alternative, but not preferably because you have to sacrifice an electrode for brain activity measures (Noreika et al., 2020), and the Cz seems especially vulnerable for artifacts introduced by arm and leg movements (Georgieva et al., 2020). In systems such as ActiveTwo, we recommend placing a combination of multiple ground and reference electrodes (CMS, DRL for online reference and mastoids for post-processing re-referencing) to get the most out of the data. This way, disturbances in one of the ground electrodes will not lead to complete removal of the participant from the study. After collection, re-referencing can be done to the coupled mastoids, one mastoid or average referencing over all electrodes. Some systems, such as Neuroscan (Compumedics, Leipzig, Germany), are using amplifiers that apply an electrical reference directly (i.e., the ability to suppress voltages common to multiple electrodes) and referencing has to be performed by post-processing average referencing only (Bigdely-Shamlo et al., 2015). And the third type of systems have extra online and offline electrodes embedded in their caps, see for example the ActiCap system (Brain Product, Gilching, Germany), which has an online reference electrode on the left mastoid (TP9) and then offline to the corresponding right mastoid (TP10, see for example in Langeloh et al., 2018).

### Parent and Infant-Friendly Laboratory

Besides the technical aspect of building a dual-EEG laboratory setup, research with infants and (young) parents requires the setting to be infant-friendly. Practically, there are a few factors that need to be taken into account when setting up your room for the experiment. Firstly, observations of direct eye contact are needed for most behavioral paradigms; therefore, we recommend to put the parent and infant on the same height. This can be accomplished by including an age-specific infant chair that can be moved upside down (e.g., Stokke Tripp Trapp). As an alternative for younger infants, a baby car chair can be put on a table that is able to be moved up or down (see for example our laboratory setting in Figure 1). Secondly, the environment for the experiment is extremely important for the outcome of the results. Therefore, one should focus on making the room as comfortable and save as possible for the parent and infant. As with any participants, the experimental room has to be neat and clean, but it also has to be baby-proof, including tidying up cables and protecting sockets. It can be helpful to make the room infant-friendly by hanging wall posters with cartoons, a changing table, placing an age-specific chair and a comfortable chair for the parent and create a comfortable place for (breast)feeding.

### Multimodal Equipment and Tools

To make use of the full potential of parent–infant EEG, we recommend collecting and integrating data from multiple modalities during real life interaction, including audio and video recordings from multiple angles. Video recordings of the session are useful to gather behavioral information and to pinpoint low-motion epochs later in the process of data-analysis (Leong et al., 2017; Wass et al., 2018; Georgieva et al., 2020; Santamaria et al., 2020). Besides neural and behavioral data on video, other biological measurements can complement research by providing information about the physiological state of both individuals during dual-EEG. Electrocardiography (ECG), for example, could be an interesting consideration, as heart rate activity is able to measure stress-related activity and can be a really easy-to-apply addition to the EEG setup.

**FIGURE 2:**
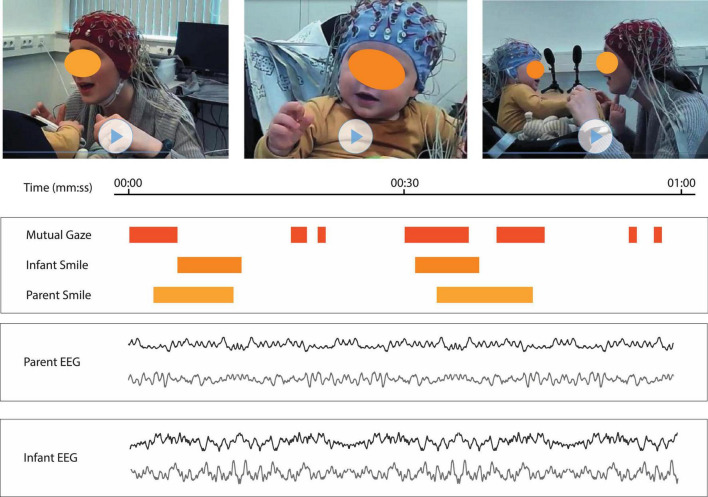
Synchronization of multimodal data and behavioral coding. Image is showing the visualization of the temporal synchronization of behavioral (video and coding) and neural (EEG) data from (a fabricated) parent–infant EEG session of 1-min-interval. On **(Top)**, three videos are displayed with mom-view, infant-view and overview. **(Middle)** Timeline and behavioral coding for mutual gaze, infant smile and parent smile are displayed. Color (orange/yellow) represents the existence of the behavior and white represents the absence of the behavior. **(Bottom)** EEG signal of two parent electrodes and two infant electrodes are displayed.

Given that a multitude of different types of data (EEG, ECG, and video) may have different but additional functional roles, it is important that all data is temporally synchronized; i.e., collected and analyzed simultaneously. It is advised to use software (e.g., non-commercial lab streaming layer or the Noldus Observer XT commercial software) that allows the integration, visualization, and processing of behavioral and neural data (example displayed in Figure 2). Correct temporal alignment of multiple datasets of different modalities is challenging but extremely important for dual-EEG studies. It is essential to add triggers to your data, to provide a cue of when a task starts, ends, or to mark certain conditions within a task. Previous parent–infant EEG research showed to be successful in synchronizing video to dual-EEG data, using a push button radiofrequency trigger signal that was sent simultaneously to both EEG amplifiers and concurrently emitted a light pulse that was visible on both video recordings (Wass et al., 2018; Santamaria et al., 2020). These start and end points of EEG and video need to be aligned before processing the data. Another way to synchronize EEG data with other modalities, such as video, is to use software that triggers the recoding of several data types simultaneously (e.g., lab streaming layer, Noldus Observer XT, E-prime, and OpenSesame).

### Age-Appropriate Designs of the Experiment

Parent–infant EEG is suited to tackle ecological validated or controlled experiments, measuring real-life social dynamics of parent and infant. However, the age of the infant who is taking part in the EEG research strongly influences the design of the experimental paradigm to maintain the quality of the research. First of all, the duration of the paradigm has to be adjusted to the age of an infant and should be planned around naps. We recommend to keep a maximum duration of ∼30 min for younger infants (<6 months) and a maximum of ∼60 min for older infants (between 6 and 18 months) for the whole session and keeping the experimental tasks to 2–3 min per condition, with short breaks in between (Noreika et al., 2020). Secondly, the interaction task between parent and infant has to be adapted to the social, motor and cognitive abilities of the infant. This can be reached by implementing age-specific paradigms and take potential developmental disorders that may influence the infant’s ability to perform the task into account. Infants can, for example, participate in a “resting-state” paradigm while watching a short movie together with a parent or participate in a task, such as, “free play” (i.e., natural interaction) (Hasegawa et al., 2016), still face paradigm (Perone et al., 2020) or a task in which the parent reads from a book. Besides parent–infant interactions, it may be interesting to conduct experiments between siblings or between infants, instead of their parents, which can provide interesting information about socio-emotional competence.

Relatedly, it is important to pre-define good baseline and control conditions for the experimental questions. Baseline conditions are usually conditions where no interaction takes place, and these periods can be used to define neural baseline activity or resting-state activity of the parent and infant brain. Resting-state activity in adults can be acquired by monitoring the brain activity while the adult is sitting still and is doing nothing, for example by closing their eyes or averted gaze. Obtaining resting-state activity in infants is more challenging, but can be achieved through various tricks (Anderson and Perone, 2018), including individual play (Benasich et al., 2008; Santamaria et al., 2020), watching an age-appropriate cartoon on a screen (without sound), gaze at a toy held by an experimenter (Wolfe and Bell, 2007), or gaze at bubbles that are blown by the parent or one of the experimenters. Another major benefit from comparing joint and individual play conditions is that it enables to control for the impact of shared external stimuli on the EEG signal (Burgess, 2013; Wass et al., 2020; Hamilton, 2021; Hoehl et al., 2021), see also Sections “Choosing a Metric for Interpersonal Coupling” and “Interpretation of the Outcome” about the problem of shared environmental input. Other control conditions can be part of an interaction where a certain type of behavior is present or missing, for example by comparing mutual gaze versus no mutual gaze, or touch by no touch.

Another point of consideration when designing the experiment is to keep in mind that your parent–infant EEG dataset will be contaminated with motion-related artifacts. You cannot simply explain an infant that it needs to sit still during the experiment. Additionally, the parent can be instructed to sit still, but you want to maintain a high level of natural interaction during your experiment, including head motion and gaze shifts toward the infant. Complete prevention of motion-related artifacts is not possible nor desirable during naturalistic parent–infant interaction, and therefore a lot of data will end up being excluded. In our experience, the extend of motion-artifacts and related data loss depends largely on the task. Tasks that elicit stress responses in the infant (e.g., crying and fussiness), such as a still face paradigm, lead to more motion artifacts than reading a book together (although animated reading can also cause many artifacts). Piloting your design for motion artifacts, testing out different instructions (e.g., “please read the book quietly and calm”), or different toys/books (e.g., some toys trigger more movement than others) is highly recommended. Still, motion-artifacts are inevitable and, therefore, we suggest to test twice the number of dyads than would be required based on standard power calculations (Noreika et al., 2020).

## Data Collection

### Best Practices in Improving Data-Quality

Parent–infant EEG data acquisition is challenging, especially with younger infants that are easily distracted, hard to instruct and have rapid fluctuations in arousal states. Sleepiness and fluctuating arousal states may influence the EEG signal and are therefore undesirable for collecting high quality parent–infant EEG data (Noreika et al., 2020). Specifically, differences in arousal states from parent and infant -that are a consequence of personal or environmental factors (i.e., bad night sleep) instead of the EEG paradigm- are a confounding factor on the brain-to-brain measurements and will affect data-quality. The state of alertness influences the spectral EEG fingerprint of adults (Ogilvie, 2001) and infants (Grigg-Damberger et al., 2007; Otte et al., 2013). For example, arousal shifts from wakefulness to drowsiness in infants from 6 to 8 months old are accompanied by a diffuse high amplitude synchronous 3–5 Hz activity, and activity that is 1–2 Hz slower than the waking EEG background activity (Grigg-Damberger et al., 2007). Many tips from infant EEG research that are described in the paper of Noreika et al. (2020) apply for parent–infant EEG research to overcome the problem of arousal, but there are additional challenges when testing infant and parent during interaction. For example, the duration of preparation can be problematic. Since you need to apply two (instead of one) caps, the infant may become tired or frustrated before you are done with the set-up. Additionally, we have noticed that parents and infants pick up stress and anxiety signals from nervous and unexperienced team members, which sometimes lead into stressed parents and over-aroused infants. Fortunately, there are several preparations you can take to speed up the preparation time and overcome frustration, high arousal states or sleepiness from either the infant or parent.

The following tips are a mix of practical instructions based on general developmental research and tips from our own parent–infant EEG experience (from two labs: Tilburg University, PI: van den Heuvel, and Interdisciplinary Center Herzliya, PI: Feldman). Good preparation and planning of the research team is needed to be as fast and precise as possible when applying the caps. This starts, for instance, by planning your lab appointment just after a nap of the infant. Parent–infant EEG cannot be conducted by one researcher alone. Instead, multiple trained researchers are needed to fulfill different roles, enabling the simultaneous application of the EEG caps and entertaining the infant. However, be careful that the room is not too crowded, that may overwhelm the infant or parent. We recommend including three researchers that are present during the setup: one project leader that has the most contact with the mom and is leading the placement of the EEG equipment, one technical assistant for EEG placement and technical preparations and control of the camera’s, computers and other equipment and one assistant that is mainly focusing on entertaining the infant. It is important that team members that will apply the cap and sensors are well trained in applying the sensors systematically and correct on subjects that are moving around. The training is also important for the team to get confidence before they start to work with infants. Capping an infant is extra challenging, because you have to work silently (e.g., sensors bumping into each other make an infant-attractive sound) and invisibly (e.g., work from behind the infant without blocking their visual field). Additionally, some EEG systems allow to prepare the EEG cap (e.g., by pre-gelling) before the participants enter the room, this will save time. It is usually best to start with the setup of the parent first, thereby you show the infant an example of what is going to happen with them and provide more time to explore the room and “warm-up.”

Generally, the steps for setting up the parent–infant EEG equipment are: (1) place sensors on the mastoids of the parent, (2) place EEG cap on the head of the parent, (3) place all sensors from frontal to occipital, (4) place additional sensors, including other physiological sensors such as ECG and, (5) repeat steps 1–4 on the infant. Clear and calm communication with the parent and infant is necessary to avoid high arousal states; stay relaxed during the communications and explain calmly what you are doing in real time. Focus on relaxing the parent, because the infant senses parental stress and this can increase the change of a fussy infant. We have noticed that food and quietly blowing bubbles really relaxes most infants. In case of very grabby infants, it is also a possibility to put a cap on top of the EEG net, but be careful with overheating the infant in case of warm room temperatures. Before you start the setup and experiment, let the infant explore the experimental room. You can also include the parent in preparing the set-up, the parent knows the infant best and is able to comfort the infant. In general, good preparation and clear communication are fundamental aspects of improving data quality.

## Preprocessing Parent–Infant EEG

### Preprocessing Software

An open available pipeline that is built for the preprocessing of dual-EEG data with infants/children (DEEP software) can been found in the recent work of Kayhan et al. (2022). Alternatively, researchers can build their own pipeline to preprocess the parent-infant EEG data. A good preprocessing pipeline for parent–infant EEG must meet two important conditions. First, it has to be compatible with a hyper-EEG dataset (i.e., parent and infant EEG in one file). Software that provides this opportunity are DEEP (Kayhan et al., 2022), Brain Vision Analyzer 2.0 (although not perfect^1^) and HyPyP (Hyperscanning Python Pipeline; Ayrolles et al., 2021) that is developed for the preprocessing and analysis for adult dual-EEG data in Python. Alternatively, software such as MNE-Python, Fieldtrip, and EEGLAB can be used to preprocess separated infant and adult data files and the hyper-EEG dataset can be made after artifact removal (see for example, the split/merge functions in HyPyP). Second, given that the preprocessing pipeline has to be suited to analyze both infant and adult EEG, the pipeline has to include multiple removal and correction software/algorithms that match infant- and adult-specific artifacts. Detailed artifact detection, reduction (e.g., filtering) and removal software are widely available for motion-contaminated epochs in adult EEG data (see for review Islam et al., 2016), but the literature and software to reduce the signal-to-noise ratio in infant EEG is limited.

Fortunately, recent papers provide some infant EEG preprocessing software that may help to develop a preprocessing pipeline for denoising parent–infant EEG data. The HAPPE and HAPPE + ER preprocessing pipelines, for instance, are great examples of MATLAB-based algorithms to clean and process continuous and event-related infant EEG data (Harvard Automated Processing Pipeline for Electroencephalography; Gabard-Durnam et al., 2018; HAPPE plus Event-Related; see preprint Monachino et al., 2021). Other recently developed EEG preprocessing pipelines include: the MADE preprocessing pipeline (Maryland Analysis for Developmental EEG; Debnath et al., 2020), the EEG-PI-L preprocessing pipeline (EEG Integrated Platform Lossless; Desjardins et al., 2021), the APICE (Automated Pipeline for Infants Continuous EEG, see preprint Fló et al., 2021), the ADJUST ICA algorithms (Leach et al., 2020), and iMARA ICA algorithms (Haresign et al., 2021b). More details about the preprocessing steps can be found in the next paragraph.

### Cleaning Data

Artifacts in general, but especially motion-related artifacts in the parent’s and infant’s EEG signal during naturalistic interaction are unavoidable, and need to be corrected or removed from the data. A study has found that the amount of EEG data that was contaminated with movement artifacts was around 95% of the total experiment time in infants (11 months of age) and adults during free interactions (Georgieva et al., 2020). Information about the amount of movement artifacts in parent–infant EEG is, however, sparse and therefore we have to be careful to draw conclusions from it. In our experience, the parent–infant EEG-data is usually less contaminated with movement artifacts and the extend of artifacts and related data loss depends largely on the design of the study and the age of the infant (see section “Age-Appropriate Designs of the Experiment”). High-motion contaminated epochs, including facial, limb and postural movements, have to be either removed from the data by employing strict rejection procedures or corrected by data-driven algorithms to separate the neural signal from motion artifacts. Analyses that differentiate and remove motion from neural data as measured during parent–infant EEG paradigms are therefore crucial to perform.

In short, a general parent–infant EEG preprocessing steps includes the following steps: (1) visually inspection of the raw data and removal of flat electrodes, (2) re-referencing the data (optional; see discussion on reference electrodes in Section “Parent–Infant EEG Equipment”), (3) filtering the data (e.g., 1–30 Hz bandpass and a 50/60 Hz notch filter), (4) interpolation or removal of spurious electrodes, (5) manual artifact rejection of high motion-contaminated segments according to the video (see for example, Leong et al., 2017; Wass et al., 2018) or visual assessment of the EEG signal by an experienced researcher, (6) data-driven algorithms for the detection of motion based on independent component analysis (ICA) or wavelet analysis, (7) manual or (semi)automatic artifact rejection to further exclude segments where the amplitude of infants’ or adults’ EEG exceeded a certain voltage (e.g., +100 μV), (8) segmentation into 2 s overlapping [epochs (1 s overlap) or 1 s epochs with 500 ms overlap (Endevelt-Shapira et al., 2021)]. Down sampling can help with reducing computational power needed to perform heavier preprocessing steps, such as ICA. However, note that choosing a too low down-sampling rate can affect high frequency analysis (i.e., gamma band). Nevertheless, using a 512 Hz rate or higher should be enough for most high frequency analyses. Also note that when data needs to be re-referenced this needs to be done after the reference electrodes are checked. This is even more important when using an average reference (using the average signal over all electrodes as a reference).

During preprocessing some important decisions need to be taken, including the question if it is best to remove bad electrodes completely from the data or to replace the signal by interpolation with neighboring electrodes. Identification of noisy or ‘bad electrodes’ that show high impedances or displacement during recording include the following type of electrode signals: no signal, a large deviation of the electrodes’ amplitude compared to the other electrodes, a relatively flat signal compared to other electrodes, high frequency noise and the lack of correlation with any other electrode (Bigdely-Shamlo et al., 2015). To keep as many electrodes/participants as possible it is ideal to interpolate a few bad electrodes with the signal of surrounding channels, but one should be aware that interpolation is the creation of dependent data and thereby might result into false neural connectivity estimates. As a general rule of thumb, some parent–infant EEG researchers have used the following rule: if <5% of the electrodes within an individual are bad in less than <5% of the group, interpolation is an accepted technique (see for example, Leong et al., 2017). How many epochs and electrodes you need from every participant to include them in the sample depends on the research question and type of paradigm. Data-selection for further analysis should be based on a minimum number of acceptable “hyper-epochs” (i.e., aligned epochs from participants 1 and 2, see Ayrolles et al., 2021), and if a dyad has less hyper-epochs than this acceptable minimum, it has to be excluded from the analyses. We recommend to at least have 8–10 artifact-free hyper-epochs per dyad (of 1 or 2 s of data), per condition. We also highly recommend to always report on this minimum and to report the average number of epochs that were retained. To keep as many participants in the study as possible, we advise to (first) closely evaluate the signal from electrodes that are known to be sensitive to movement. For example, a recent infant EEG study showed that especially electrodes C3, CP2, CP5, Cz, P7, T7, and TP9 are high-motion contaminated electrodes in infants during free play (Georgieva et al., 2020). Alternatively, one is advised to remove electrodes and segments that show a spurious signal in many participants of the study. Parent–infant studies so far, seem to follow this strategy by including between two (Leong et al., 2017; Wass et al., 2018) and sixteen (Santamaria et al., 2020) electrodes for further analysis.

There are several approaches of data cleaning needed to distinguish neural data and physiological and non-physiological artifacts. Different cleaning techniques have their own benefits and pitfalls. For example, visual assessment and removal of data significantly improves data quality, but it is time consuming and inter-observer variability might introduce subjective, biased variation in the data. Beside visual assessment of the EEG signal, applying an automatic algorithm to detect noisy segments might be an option for dual-EEG processing as well, such as MNE’s “AutoReject” with Bayesian optimization as the threshold method (Djalovski et al., 2021; Endevelt-Shapira et al., 2021). Autoreject is an automatic data-driven algorithm for detection and repair of bad segments, using optimal peak-to-peak rejection thresholds subject-wise (Jas et al., 2017). The Autoreject algorithm removes trials containing transient jumps in isolated electrodes, but does not work well for systematic physiological artifacts that affects multiple electrodes. Another important approach of data-correction is independent component analysis (ICA). ICA has its imperfections, such that removing the ICA components partially distorts the data and may potentially impact the sensitivity of later analyses (Dimigen, 2020), but it generally works great for data-correction and also provides a major opportunity to limit the amount of data loss. In general, ICA is useful for extremely stereotypic motion, such as eye blinks in adults, but is more limited when processing infant EEG data due to the less stereotypical nature of those movements (Fujioka et al., 2011; Georgieva et al., 2020; Noreika et al., 2020). It is recommended to add ICA as an additional preprocessing step, specifically to correct for stereotypical movement artifacts and to limit data loss as a consequence of strict rejection procedures. For example, semi-automatic ICA, such as MNE’s implementation of FastICA and CORRMAP (Viola et al., 2009), seems to work well with oculomotor artifacts in parent–infant EEG (Endevelt-Shapira et al., 2021). The CORRMAP algorithms are based on the idea that stereotypical artifacts are generally similar over large number of participants from the same sample and age (e.g., parents or infants). CORRMAP works as follows: the code allows to manually select an independent component (IC), eye blinks for example, for exclusion in one participant. This chosen component serves as a template for selecting and excluding similar (highly correlated) components in other participants. Most software algorithms are generally not trained on infant EEG and therefore ICA is usually not good enough to detect and correct most motion artifacts in infant EEG.

However, newly developed automatic ICA (iMARA; see Haresign et al., 2021b; ADJUST; see Leach et al., 2020) and wavelet-thresholding software combined with MARA (HAPPE + ER; see preprint Monachino et al., 2021) for infant EEG are promising tools for isolating and correcting artifacts. At this stage, iMARA and ADJUST ICA algorithms are trained on and verified for, respectively, continuous EEG and ERP of infants that are between 10 and 12 months old and ERP of infants between 4 and 6 months old. As a result, the algorithms may work less well on EEG data collected at different age ranges or under different conditions. We therefore recommend to visually inspect and verify selected independent components, especially in EEG data from infants with another age range than those that are verified in the papers. Additionally, it is recommended to visually inspect and verify randomly selected segments for excessive noise in the EEG signal after all automatic removal and correction steps. We believe that combining manual assessment of data cleaning with automatic algorithms and visual inspection of the outcome result is of the utmost importance. Comparing different EEG cleaning techniques will be paramount in learning what the best options are for parent–infant EEG. Altogether, cleaning parent–infant EEG data is time consuming and multiple visual inspections and (semi-)automatic techniques are needed to remove and correct artifacts from EEG data. Researchers should be aware that many data will get lost, since you need good data from both parent and infant. Finding a balance in removing enough movement-contaminated data from the actual neural data and prevent overcorrection is challenging (Fujioka et al., 2011).

## Analyzing Data

### Techniques for Interpersonal Behavioral and Neural Coupling

Parent–infant EEG enables the rich collection of behavioral and neural information of the two participants, making all types of neurobehavioral coupling analyses possible. Behavioral parameters have important purposes in the analysis. More specifically behavioral information should lead the decision on which EEG segments are going to be analyzed and which are excluded. First, behavioral information may help to select low-motion contaminated EEG data by identifying and removing periods with high motion artifacts such as jaw and limb movements. Second, behavioral coding provides the opportunity for neurobehavioral coupling by labeling each EEG-epoch with the behavioral label of interest (e.g., mutual eye contact versus no eye-contact; joint play versus individual play; affective touch versus no touch). Moreover, microanalytic coding (i.e., intervals of 1 s or even per frame) and global analytic coding (i.e., minutes or session wise) of the behavior enables the neurobehavioral coupling of specific behavioral states, social cues, or task-conditions of interest. Microanalytic coding usually includes micro-behaviors, such as eye gaze, vocalizations, while global analytic coding usually includes broader concepts, such as maternal sensitivity and mutual interactions.

Analytic scores are generally based on conditions such as 0 (not apparent) and 1 (apparent) or on Likert scales. See Figure 2 to get a basic idea of possible labels during a free interaction (“free play”) experiment. There are many behavioral coding systems that have been developed for parent–infant interactions that have been used in behavioral synchrony papers. See for example, the MACY system that distinguishes between ‘positive and negative parenting’ based on a large set of maternal, infant, and dyadic rating scales for scoring multiple qualitative dimensions of interactive behavior (Boeve et al., 2019), the Coding Interactive Behavior (CIB) for a rating scale of parent, infant, and dyadic affective states and interactive styles (Feldman, 1998; Endevelt-Shapira et al., 2021), the Infant regulatory and maternal regulatory scoring system (IRSS and MRSS) for facial affect, direction of gaze, vocalizations, gestures, leaning, touching, self-comforting, distancing and stress (Weinberg et al., 2008), maternal sensitivity and attachment (Beebe and Steele, 2013), and attention, affect, orientation, touch, and composite facial-visual engagement (Beebe et al., 2016). Other interesting (and more basic) parameters during parent–infant interactions can be measured as well: infant-directed speech (Shruti et al., 2018; Kalashnikova et al., 2020), infant vocalizations (e.g., cry, fuss, yawn, and positive vocalizations, Feldman et al., 2010b), and dyadic coordination of gaze (Northrup and Iverson, 2020). These behavioral coding schemes and labels are just a few examples of the enormous number of possibilities that facilitate all kinds of behavioral coding available during parent–infant interaction. Choosing the best coding-scheme can therefore be difficult. The most important criterium for the coding scheme is that it has to be adapted to the research questions of interest. If one, for instance, is interested in how parental touch may induce brain-to-brain synchrony, it is important to choose if you are interested in the duration (Moreno et al., 2006), the number of instances (Reece et al., 2016), or in the quality of touch (Feldman et al., 2010b; Brzozowska et al., 2021). Additionally, it might be interesting for this research question to include conditions such as maternal touch during mutual gaze versus maternal touch during infant’s averted gaze (see for example, Feldman et al., 2010b), then gaze is also important to code. It is crucial to note that behavioral coding can be time consuming and each extra label means that you have to go over the video one more time, a good trade-off between enough and not too many labels is therefore essential.

### Choosing a Metric for Interpersonal Coupling

**FIGURE 3:**
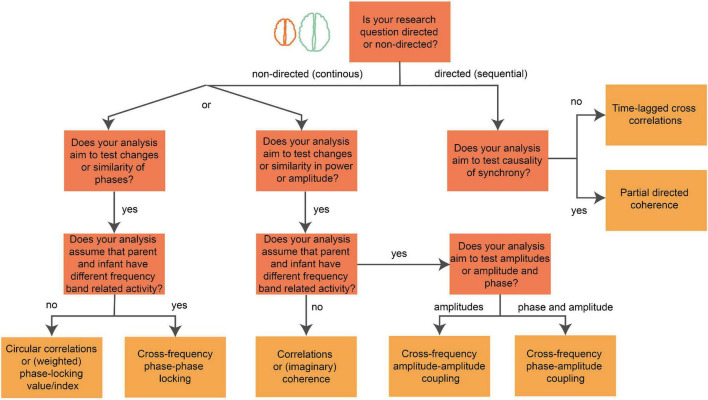
Decision tree of brain-to-brain coupling analysis. Image showing steps of a decision tree for the interpersonal coupling analysis after preprocessing. This flow-chart supports in finding a metric that is in line with the research question. Each dark-orange node represents a question about the research question (e.g., “Does your analysis aim to test causality of synchrony?”), each connection represents the answer (e.g., “yes” or “no”) or shows a choice between options (e.g., “or”), each yellow node represents a single or various coupling metrics (e.g., Partial directed coherence) that are in line with the research question. The decision tree starts at the orange node with the text: “Is your research question directed (sequential: time-lagged or causal) or non-directed (continuous in synchrony)”?

The level of brain-to-brain coupling from parent–infant EEG data can be estimated through various methods and even more corresponding metrics (Czeszumski et al., 2020). Different techniques answer different aspects on the interpersonal coupling. Finding the best-method to run the data is therefore crucial for the outcomes and the interpretation of the results (Hamilton, 2021). It is challenging to determine which interpersonal connectivity variable provides the most valuable information about the relationship between parent and infant for a specific research question. Figure 3 shows a decision tree for the (postprocessing) analysis of parent–infant EEG that may help picking the method of choice. One can explore the analyses options with our open available parent–infant Hyper EEG dataset (example data can be downloaded from our OSF page^2^). A description of our open available parent–infant EEG dataset can be found in Figure 1. We used Brain Vision Analyzer 2.0 to preprocess the data and MNE and HyPyP (Ayrolles et al., 2021) to compute and visualize PLV values of intra- and interbrain connections.

The amount of research questions that can be analyzed from neurobehavioral coupling during parent–infant free play interactions are limitless. The first step (Figure 3) of choosing the best method is by deciding whether the research question is about *concurrent* (synchronous or non-directed) or *sequential* (causal, time-lagged, or directed) interpersonal neurobehavioral coupling during social interaction. Synchronous coupling is the method of choice when the research outcome is expected to be the same in both social partners, for example, if X (e.g., the parent EEG) is high than Y (e.g., the infant EEG) is also high (Wass et al., 2020). This approach includes the computation how behavior and neural activity covary or correlate within the dyad during a specific condition or during the whole experiment. Here, we will give a few examples for synchronous coupling within free play paradigms: ‘Does overall more parental touch during the experiment leads to enhanced brain-to-brain coupling,’ ‘Does the group with more positive parenting scores show enhanced brain-to-brain coupling?’, ‘Does the amount of infant-directed speech influence alpha power in infants, and, does the quantity of vocalizations from the infant influence alpha power in mom?’ (*concurrent*, brain-to-behavior coupling). Sequential coupling is the method of choice when the research question(s) include a (causal or forward/backward) prediction on activity or behavior within the dyad, for example, the hypothesis that X (e.g., the parent EEG) influences the signal of the social partner Y (e.g., the infant EEG) over time (Wass et al., 2020). In the sequential coupling method, the timescale and direction of the relationships are important to include in the research question. Examples of research questions that include sequential coupling methods are: ‘Does parental theta power positively predict infant theta power during joint play?’, ‘Does infant gaze directed to mom leads to changes in the mom’s EEG, and does it forward-predict changes in the infant’s EEG?’.

Globally, *concurrent* and *sequentia*l relations can be computed from different aspects of the signal (Figure 3): power/amplitude and phase (Haresign et al., 2022). Therefore, the next step of choosing the best method is to determine which part of the signal is important for the research question. First, power correlations and coherence measures are usually used for measuring the similarity between cognitive states of two individuals that remain stable over a larger time window (Ayrolles et al., 2021). For instance, EEG power represents the amount of activity in a certain frequency range and is linked to different brain functions, such as control of behavior in social situations with substantial attentional (Orekhova et al., 2006) and emotion load (Allen et al., 2018). In parent–infant EEG research, fluctuations in theta power are linked to changes in shared attention during solo and joint play of parent and infant (Wass et al., 2018), enhancement of alpha and theta power in both parent and infant are linked to changes in directed gaze (Leong et al., 2017) and fluctuations in alpha band (e.g., fluctuations in alpha asymmetry) are linked to changes in emotional states of mother and child (Atzaba-Poria et al., 2017; Krzeczkowski et al., 2020; Perone et al., 2020; Santamaria et al., 2020). Second, phase synchronization of neural oscillations (i.e., the interpersonal synchronization of oscillatory signatures) is the most common alternative for power correlations in the analysis of dual-EEG. Phase-locking measures have a finer-grained account of timing and have been used to measure fluctuations of ongoing cognitive processing on a smaller timescale (Ayrolles et al., 2021). Parent–infant EEG studies so far showed that brain waves (phases) of mother and infant are synchronized to each other and that this synchronization is modulated by emotional valence (Santamaria et al., 2020) and maternal chemosignals (body odor) (Endevelt-Shapira et al., 2021). Moreover, enhanced phase-locked brain-to-brain synchrony predicts higher infant social learning likelihood (preprint, Leong et al., 2019). A review of existing parent–infant EEG studies so far is beyond the scope of this guideline paper, please see the reviews of parent-child EEG for an extensive overview of the conditions, findings and possibilities of parent–infant EEG for developmental research (Markova et al., 2019; Wass et al., 2020; Turk et al., 2022).

The next step of the analysis is to decide which connectivity or coupling method is the best for your data (Figure 3). There are many methods to compute brain-to-brain and interpersonal brain-to-behavior coupling and there is no golden standard yet (Turk et al., 2022). Common analysis techniques in the dual-EEG field to compute *concurrent* (non-directed) brain-to-brain or brain-to-behavior coupling are: correlations (Corr) of power/amplitude and coherence-based (Coh) measures of power/amplitude in a specific frequency range (Atzaba-Poria et al., 2017; Zamm et al., 2018; Krzeczkowski et al., 2020). Non-directed phase synchrony can be measured through phase-locking value (PLV) (Santamaria et al., 2020), (weighted) phase-locking index (PLI or wPLI) (Sanger et al., 2012; Endevelt-Shapira et al., 2021), circular correlation coefficients (CCorr) (Burgess, 2013), and subsequently by graph analysis to explore functional network within and between dyads (Sanger et al., 2012; Santamaria et al., 2020). *Sequential* brain-to-brain coupling can be computed using Granger causal relations or relations with a specific lag. Common measures including time-lagged cross correlations (Wass et al., 2020), and partial directed coherence (PDC) (Leong et al., 2017; Santamaria et al., 2020) are methods to estimate if certain brain activity of one brain forward (or backward) predicts the neural activity response of the social partner. PDC is a directed connectivity measure reflecting a frequency-domain representation on the concept of Granger causality (GC) (Baccalá and Sameshima, 2001). Specifically, PDC is calculated from the multivariate autoregressive (MVAR) coefficients which are generally obtained from fitting autoregressive models to the ‘raw’ power/amplitude data in a specific frequency domain (Baccalá and Sameshima, 2001). PDC or GC can be used to test a hypothesis at a large time-scale (e.g., whole conditions): the hypothesis that X (e.g., the parent EEG) is Granger causal of Y (e.g., the infant EEG), by showing that a combination of X and Y better predicts Y than X would predict Y alone. At the fine timescale, time-lagged cross correlations are a better alternative to estimate if X (e.g., certain micro behavior or neural correlate of parent) predicts Y (e.g., behavioral or neural response of infant), because GC excludes information on micro behavioral or neural fluctuations over time (Novembre and Iannetti, 2021). It is important to note, however, that time-lagged cross correlations are not measuring causality and if causal relations are hypothesized then PDC should be used to estimate the directed relationships between participants.

Furthermore, all connectivity and coupling metrics are in some extend vulnerable for detecting false-positive connections, but some show to be more robust and may therefore be the metric of choice for outside laboratory conditions. Studies from the past few years used dual-EEG to test brain-to-brain synchrony “in the wild,” outside the laboratory, during a variety of naturalistic interactions; in daily conversations, classroom settings (Dikker et al., 2017), museums and festivals (Dikker et al., 2021), or sports (Stone et al., 2019). Several authors have argued that for research using ecologically-valid paradigms in non-controlled settings, the classical methods such as phase-locking value, correlation and coherence are not the methods of choice (Burgess, 2013). One of the methodological issues that have been discussed regarding the use of dual-EEG metrics is that they may ‘detect’ *changes* in synchrony in response to shared sensory stimuli – a risk that is greater in noisy, uncontrolled settings outside the lab (Burgess, 2013; Wass et al., 2020; Hamilton, 2021; Hoehl et al., 2021). Specifically, Burgess (2013) showed that false-positive phase synchrony-based hyper-connections arose between pseudo-pairs from different people independently measured, but under the same experimental conditions. There are several measures that avoid (in a larger scale) false-positive hyper-connections. For example, in an extensive qualitative analysis, the author show that CCorr is less (as compared to PLV for example) susceptible of picking up spurious hyper-connections (Burgess, 2013). CCorr is the alternative correlation metric for circular data and measures the circular covariance of differences between the observed phase and the expected phase (i.e., the phase variance) (Burgess, 2013). Along with this, in a recent infant-adult dual-EEG study using face-to-face free interactions, to avoid spurious hyper-connections that could be result of similar sensory experiences of the participants, the authors decided to use wPLI (weighted extension of PLI) as their measure for inter-brain phase synchrony (Endevelt-Shapira et al., 2021). By weighing each phase difference according to the magnitude of the lag, phase differences around zero only marginally contribute to the calculation of the wPLI. This procedure reduces the probability of detecting false-positive hyper-connectivity in the case of noise sources (shared environmental inputs at the perceptual level) with near-zero phase-lag and increases the sensitivity in detecting phase synchronization. Finally, imaginary coherence (ICoh, i.e., the absolute value of the imaginary part of coherence) seems to be a more robust alternative for coherence-based measures of intra-brain connectivity (Nolte et al., 2004) and brain-to-brain synchrony in relatively noisy settings (Dikker et al., 2021). Specifically, ICoh is calculated by computing the spectral density (power) of each participant and the cross spectral density between them, to describe the average phase difference and consistency of the phase difference (Dikker et al., 2021). The end result of the computation is a complex number to describe the coherence, including a real part representing how much of the coherence is driven by instantaneous interactions and an imaginary part that shows how much of the coherence is based on lagged interactions (Nolte et al., 2004; Dikker et al., 2021). Choosing a more robust metric, such as CCorr or ICoh, might result in less false positive estimations of interpersonal synchrony when studying dual-EEG outside the lab.

The next challenge of the analysis lies in choosing the frequency band-width for the computation of interpersonal brain-to-brain coupling in the power band of interest. It is important to note that different frequency ranges have different functional roles in the brain and that frequency peaks differ between infant and adult (Noreika et al., 2020). Specifically, frequency peaks are increasing over age due to neurodevelopmental changes (Orekhova et al., 1999; Marshall et al., 2002), and therefore, EEG power spectrums of parent and infant show large differences (Noreika et al., 2020). For instance, alpha power has a peak frequency from 6 to 9 Hz in infants (from 5 months to 4 years of age, Marshall et al., 2002), while adult alpha peaks between 9 and 12 Hz (Hill et al., 2020). A problem related to the different frequency band activity of parent and infant is the fact that the slower nature of infant neural responses might cause a delay in the establishment of interpersonal neural relations between adult-infant dyads as compared to adult-adult dyads. Slow infant EEG activity might affect the timescale in sequential analysis, which makes it essential to optimize the temporal resolution (i.e., the lag) of the analysis. A smart technique for temporal and frequential optimization in parent and infant has been shown in the parent–infant EEG paper of Wass et al. (2018), who showed that optimization can be reached by selecting the peak cross-correlation value from all computed cross-correlations for different time-lags and power of a whole frequency range. Many interpersonal measures estimate interpersonal coupling within identical frequency bands, which can be problematic when comparing the EEG time series of adults and infants, because it forces you to decide which peak frequency is guiding. Parent–infant EEG studies have focused mainly on the infant’s frequency band (e.g., Leong et al., 2017; Santamaria et al., 2020), while it would be interesting to look at both spectra using a cross-frequency method (Haresign et al., 2022).

Cross-frequency coupling overcomes the problem of measuring synchronization across different dominant frequencies, which makes them valuable metrics for parent–infant EEG (Noreika et al., 2020). Cross-frequency coupling is possible through (1) *phase-phase*, measuring the quantity of phase-locking across frequencies of parent and infant, (2) *phase-amplitude*, measuring if amplitudes in specific frequencies in the infant are coupled to the phase of an oscillation in the parent, and (3) *amplitude-amplitude coupling*, measuring the correlation of frequency power in their age-specific frequency band (Canolty and Knight, 2010; Palva and Palva, 2018). The first option, cross-frequency *phase-phase* coupling (i.e., cross-frequency phase-locking), provides a method for linking activity that occurs at significantly different frequency rates (Canolty and Knight, 2010), and might therefore be a more appropriate method to measure phase-locking between adult and infant EEG (Haresign et al., 2022). The DEEP pipeline includes scripts to compute cross-frequency PLV between adult and infants (Kayhan et al., 2022). A dual-EEG dataset and a full pipeline to measure a wide variety of event-locked EEG changes in child-adult neural connectivity can be found on Github (see preprint, Haresign et al., 2021a). The second option, cross-frequency *phase-amplitude* coupling, provides the opportunity to examine research questions that address the different operating frequencies in the adult and infant brain, and thereby reveal possible differences in functions. For example, by examining the possible modulating role of parental low-frequency oscillations on the infant’s amplitudes of higher frequencies during joint play. Although not implemented in parent–infant research, a recently proposed method of time-frequency based cross-frequency *phase-amplitude* coupling (the time-frequency phase-amplitude coupling software, t-f PAC) of Munia and Aviyente (2019) seems promising for the implementation in dual-EEG with parent–infant dyads. Specifically, the t-f PAC method is built to measure coupling strength between different frequencies by estimating phase and envelope of low and high frequency oscillations and is more robust to address varying signal parameters (Munia and Aviyente, 2019). The third option, cross-frequency *amplitude-amplitude* coupling, provides a method to correlate amplitude envelopes of distinct (e.g., slow and fast) frequency bands without taking the phase into account (Palva and Palva, 2018). To date, cross-frequency coupling is largely unexplored in parent–infant EEG. We believe that the implementation of this method will provide many exciting opportunities and might reveal new interbrain discoveries in future studies.

### Statistics and Robustness of Results

Differences between conditions within-dyads (e.g., during joint gaze versus averted gaze) or groups (e.g., brain-to-brain synchrony of dyads with anxious or non-anxious parents) can be analyzed with a *t*-test or ANOVA. If assumptions of independency of the data are not met (i.e., non-normally distributed data) than generalized linear mixed models (GLMM) are a better alternative to perform the analysis (Nguyen et al., 2021a). GLMM is comparable with an ANOVA, but estimates fixed and random effects, in which the linear predictor contains random effects in addition to the usual fixed effects. GLMM is especially suited for the analysis of dual-EEG and multimodal data, because it allows for the integration of different types of behavior and interacting brains in one analysis-model (see for review, Hamilton, 2021). GLMM has been adopted by parent-child fNIRS studies (not by parent–infant EEG studies so far). For instance, Nguyen et al. (2021b) used GLMM to show that turn-taking between child and parent was predictive for neural synchronization during the conversation over time.

Due to the possibility of detecting spurious connections, it is strongly recommended to include covariates, control conditions and sensitivity analyses to validate the robustness of the results (Burgess, 2013; Hamilton, 2021; Hoehl et al., 2021). Small variations in sample conditions, experimental conditions and analysis methods can influence the outcome and have to be taken into account as covariates. For example, variations in infant age and the accompanied developmental shifts in spectral activity (Anderson and Perone, 2018) or low and high arousal states of the infants (Noreika et al., 2020) could influence the connectivity strength. Additionally, environmental conditions, such as smartphones, air-conditioning and the presence of another person in the experimental room diminishes oscillatory behavior (Rolison et al., 2020). It is therefore important to exclude EEG of the dyads with altered environmental or experimental conditions from the sample. Sensitivity analyses can be built into the research design using control groups, for instance by comparing mom and infant brain-to-brain coupling versus adult stranger and infant brain-to-brain coupling, if one is interested in caregiver-specific interactions or synchrony (Endevelt-Shapira et al., 2021). A more common method is non-parametric permutation testing by creating random pseudo-dyads or pseudo-conditions from the sample to compare real pairs/conditions versus a distributions of pseudo pairs/conditions (Burgess, 2013; Ayrolles et al., 2021). Specifically, a permutation test is a type of statistical significance test in which a distribution of connectivity measures is obtained by calculating all possible values between conditions or groups by randomizing time (EEG epochs) or by randomizing pairing of participants (pseudo-dyads).

When running multiple tests for hypothesis-driven analyses, it is recommended to implement false discovery rate (FDR) correction to control for multiple comparisons when comparing the averages of a group or a condition (i.e., in which the frequency, the specific electrodes and the timing or lag has been selected). In contrast, FDR is usually not suitable for exploratory analysis, in which researchers conduct many different tests to explore different frequencies, spatial and/or timing points. Relatively stringent multiple correction procedures such as FDR are based on the number of tests and may fail to detect true effects in exploratory analysis. Non-parametric permutation testing is often considered the best correction method for exploratory analysis since it uses a distribution derived from permuting the observed scores, rather than assuming that the population has a specific distribution, for instance, a normal distribution. FDR and permutation tests, which are built in the pipelines of MNE-python and HyPyP, allow for the calculation of intra and inter connectivity measures by randomizing conditions (in time) or pairing of participants (pseudo dyads or pseudo groups) (Ayrolles et al., 2021).

## Data Interpretation

### Interpretation of the Outcome

The final stage of this guideline assists with the interpretation of the findings. In some situations, brain-to-brain coupling is mostly driven by shared cognitive and affective processes, as opposed to more natural and interactional situations where simultaneous motoric behavior (synchrony in motoric behavior) and shared environmental input have a bigger influence on brain-to-brain coupling (for a discussion on the neural pinnings of brain-to-brain synchrony see reviews: Markova et al., 2019; Wass et al., 2020; Hamilton, 2021; Turk et al., 2022). The interpretation of brain-to-brain coupling outcome includes more than simply describing enhanced or reduced brain-to-brain synchronization in certain groups or conditions. It is important to understand that alternative interpretations of enhanced/reduced brain-to-brain synchrony need to be considered as other factors such as behavioral synchrony and shared environmental stimuli could drive synchrony as well.

Parent–infant interaction is characterized by coordination of behavior and brain-to-brain synchronization (Markova et al., 2019), making motoric synchronization an important factor to implement in the research and interpretation, even in low-movement interactional conditions when subjects are sitting still. For example, eye movement and other micro-interactions are closely linked to social interaction and thus to neural data of interest (Leong et al., 2017; Wass et al., 2018). This suggests that synchronization in motor behavior and facial affect (e.g., raising brows simultaneously) may drive brain-to-brain synchrony as well. A significant amount of the movement artifacts will be reduced after proper data cleaning and sensitivity analyses. However, there always remains a risk that motion-contaminated data may drive EEG power, phase estimations and intra- and interpersonal correlations (Burgess, 2013; Noreika et al., 2020; Hamilton, 2021). An approach to reduce the signal-to-noise ratio is by analyzing only specific frequency bands that are less sensitive for motion. High-frequency activity, such as gamma-band activity, shares many spatial, temporal and spectral properties with muscle artifacts in adults (Muthukumaraswamy, 2013) and is therefore more at risk for being contaminated with motor synchrony. Unfortunately, information about spectral signatures of infant movements is largely undocumented. Most reliable frequency bands in infant EEG are baby-alpha (6–9 Hz) and theta (3–6 Hz) (van der Velde et al., 2019; Haartsen et al., 2020). Moreover, a small cohort study (*N* = 12) showed that infant jaw and arm movements are related to increased beta power (∼15 Hz), but also baby-alpha and theta power are decreased for all (jaw, hand, arm, foot, and leg) motion types (Georgieva et al., 2020). Selecting only motion free epochs in baby-alpha and theta band could therefore result in enhanced power spectra of both partners and thus in enhanced brain-to-brain synchrony. An approach would be to embrace motoric synchronization of parent–infant interaction and adopt this measure of synchronization into the statistical model, for example to show that motoric synchronization drives brain-to-brain coupling as well as the internal cognitive processes (Kruppa et al., 2020; Wass et al., 2020; Hamilton, 2021). For pure measures of brain-to-brain synchronization it is important to minimize alternative interpretations, for example by filter out as many movement artifacts as possible from the EEG data.

The second interpretation challenge is the problem of shared environmental input that could drive the amount of brain-to-brain synchrony (Burgess, 2013). This interpretation challenge of dual-EEG (and hyperscanning in general) is extensively discussed in a recent review of Hamilton (2021). There is not a single solution to encounter this problem, but integrating multimodal data into the theoretical framework of parent–infant EEG analysis (e.g., by implementing mathematical models to predict brain, behavioral and physical correlates in both individuals using general linear modeling or the mutual prediction theory) seems to have the highest potential (Hamilton, 2021). In all, we believe that naturalistic experimental conditions, sophisticated statistical models and validation, significantly reduces the problem of shared environmental input and motoric synchrony as potential drivers of brain-to-brain synchronization, but do not complete rule them out. Behavioral and neural connectedness, as well as the shared environment are all part of parent–infant synchrony and therefore being honest about the strength and flaws of the dataset, design and analyses is absolutely necessary for the interpretation of the research.

## Concluding Remarks

The rapidly evolving field of parent–infant EEG is exciting and brings many opportunities to study novel interpersonal neurobehavioral correlates. To accommodate the growing demand for open parent–infant EEG databases and transparent methodological pipelines, this article aimed to support scientific progress by making our methods and approach openly available and showcase a guideline on how to implement, run, and analyze parent–infant EEG paradigms during natural interactions. Moreover, we reviewed and have provided practical tips and tricks that tackle the challenges that one may encounter during preparation of the experimental setup, data-collection, preprocessing of dual-EEG, analyzing brain-to-behavior and brain-to-brain coupling and interpretation of the outcome. The parent–infant EEG guideline provided here will facilitate standardized dual-EEG analyses. We hope to support the scientific progress of parent–infant EEG to replicate former results, and help in the discovery of new interpersonal neural correlates of the developing social brain.

## Author Contributions

ET, JL, and MH contributed to conception and design of the article. ET, JL, MH, YE-S, and RF wrote sections of the article. All authors contributed to manuscript revision, read, and approved the submitted version.

## Conflict of Interest

The authors declare that the research was conducted in the absence of any commercial or financial relationships that could be construed as a potential conflict of interest.

## Publisher’s Note

All claims expressed in this article are solely those of the authors and do not necessarily represent those of their affiliated organizations, or those of the publisher, the editors and the reviewers. Any product that may be evaluated in this article, or claim that may be made by its manufacturer, is not guaranteed or endorsed by the publisher.

## Footnotes

1 Brain Vision Analyzer can be used for preprocessing and analyzing dual-EEG data, but some issues need to be prevented. For example, a dual-EEG dataset contains all electrodes twice. This can give errors when interpolating your data or running ICA based on electrode positions. By renaming your channels, e.g., Cz-Baby and Cz-Mother, you can prevent this issue from happening.

2
osf.io/krdn5/
